# Supplementation with rumen-protected proteins induces resistance to *Haemonchus contortus* in goats

**DOI:** 10.1038/s41598-018-37800-3

**Published:** 2019-02-04

**Authors:** S. Cériac, H. Archimède, D. Feuillet, Y. Félicité, M. Giorgi, J.-C. Bambou

**Affiliations:** 1 URZ, Unité de Recherches Zootechniques INRA, 97170 Petit-Bourg, Guadeloupe France; 2 PTEA, Plateforme d’Expérimentation sur l’Animal INRA, 97170 Petit-Bourg, Guadeloupe France

## Abstract

Resistance to gastro-intestinal nematode (GIN) in small ruminant is expected to arise from protein-rich rather than from energy-rich feeds. The objective of this study was to investigate the effect of the quality of the dietary proteins on the response of Creole goats to *Haemonchus contortus*. Three diets were compared: no supplementation (Hay: hay *ad libitum*), Control supplement (CS: hay *ad libitum* +2% BW of CS at 70 g of by-pass proteins/kg) and supplement enriched in rumen-protected proteins (RPP: hay *ad libitum* +2% BW of RPP at 139 g of by-pass proteins/kg). The FEC (faecal eggs counts) and the TFEC (total faecal eggs excreted/day) were significantly lower in the RPP. No difference was found between the supplemented diets for the total number of nematodes, but the RPP reduced the parasite prolificacy. The highest IgA responses were observed in animals with the highest nematode burden (Hay compared with CS diets). However, while the FEC and the TFEC were lower in animals feed with the RPP the IgA response were similar to those of the Hay. The IgA response that control GIN egg production in sheep could be one mediator of the resistance to *H. contortus* induced with by-pass proteins in goats.

## Introduction

Small ruminant breeding, like other livestock production systems, now faces the major challenge of increasing its output with fewer resources by environmentally-friendly practices. In addition to these constraints, internal parasites among which gastrointestinal nematodes (GIN) threaten small ruminant husbandry throughout the world due to the evolution of anthelmintics resistance, the mainstay of current treatments^1,2^. Furthermore, the recent knowledge about the environmental side-effect of anthelmintic residues and the issues of public health about chemical residues in animal products reinforce the need to develop additional control strategies for sustainable production^3,4^. Three of the most promising methods which aim at enhancing the host immune response are exploitation of genetic resistance, potentially vaccination and nutritional supplementation.

In small ruminants, numerous studies have shown that the nutritional status affects significantly the host response against GIN infection^5^. Indeed, it has been suggested that an improved nutritional status would fulfil the increasing needs in proteins and calories of the immune response for the production of immune cells, mediators and the repairing of damaged tissues to face invading pathogens^6–8^. In small ruminants, numerous studies have shown that nutrient supplementation improve either the resilience or the resistance to GIN infections^9,10^. The respective impact of metabolizable energy or proteins on the host response to GIN has long been discussed in ruminants because metabolizable energy supplementation induces metabolizable proteins supply from the ruminal microbial synthesis. Indeed, in ruminant intestinal proteins derived from both dietary proteins escaping ruminal degradation (i.e. by-pass protein) and microbial proteins synthesized in the rumen. Nonetheless, Houdijk concluded in a literature review, that it is likely that the host response would be more sensitive to moderate metabolizable protein scarcity than metabolizable energy^11^. The poorer compatibility in term of amino acid composition of microbial proteins to the needs of the immune response compared with by-pass protein would explain this difference. Since high level of protein supplementation is not an option when production efficiency is an objective, manipulation of dietary protein that affects the quality of intestinal proteins is a key step for fine-tuning of nutritional strategies for a better control of GIN.

## Results

### Zootechnical, nutritional and parasitological parameters

**Figure 1:**
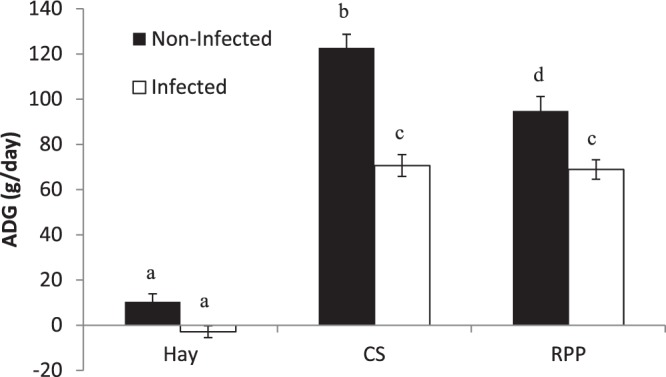
Average daily gain means (ADG) of Creole kids according to the diets (Hay^1^, CS^2^, RPP^3^) infected with an oral single dose of 10,000 third-larvae stage (L3) of *Haemonchus contortus* or non-infected. Different superscripts indicate differences between least square means at *P* < 0.05. ^1^Hay distributed *ad libitum* non supplemented. ^2^CS, Control Supplement, Hay distributed *ad libitum* + CS 2% of BW. ^3^RPP, Supplement enriched in Rumen Protected Proteins, Hay distributed *ad libitum* + RPP 2% of BW.

The composition and nutritional values of the diets are shown in Table 1. In the non-infected groups the highest average daily gain (ADG) was observed for supplemented animals (CS and RPP) (Fig. 1). The ADG was higher for the animals fed with the CS diet compared with the RPP (*P* < 0.001). A significant weight loss was observed during the experimental infection for animals fed with Hay (−2.85 g/day, *P* < 0.001). No difference was observed between the infected animals in the CS and the RPP groups. However, the reduction of the ADG between infected and non-infected animals was higher in the CS group compared with the RPP (−42.4% *vs*.−27.3%, *P* < 0.05).

**Table 1:** Nutritional values of the diets.

Chemical composition	Diets		
	Hay^1^	CS^2^	RPP^3^
CP^4^ (%)	8.1	23.3	23.3
DCP^5^ (%)	5.0	18.9	19.3
PDIA/kg^6^	24	70	139.3
PDIN/kg^7^	66	160.9	182.8
PDIE/kg^8^	51	126.5	174.5
FU/kg^9^	0.6	0.98	0.99
RDS^10^ (%)	—	61.1	37.8
RDN^11^ (%)	49	68.3	33.5

^1^Hay distributed ad libitum.

^2^CS, Control Supplement, Hay distributed ad libitum + CS 2% of BW.

^3^RPP, Supplement enriched in Rumen Protected Proteins, Hay distributed ad libitum + RPP 2% of BW.

^4^CP: Crude Protein.

^5^DCP: Digestible Crude Protein.

^6^PDIA: Dietary protein undegraded in the rumen, but truly digestible in small intestine.

^7^PDIN: Amount of microbial protein that could be synthetized in the rumen from the dietary nitrogen when energy and other nutrient are not limiting factors.

^8^PDIE: Amount of microbial protein that could be synthetized in the rumen from Energy available in the rumen when nitrogen and other nutrient are not limiting factors.

^9^FU: Feed Unit (Net Energy).

^10^RDS starch: Rumen Degradable Starch.

^11^RDN nitrogen: Rumen Degradable Nitrogen.

A significant effect of the supplementation was observed for 6 out of the 8 blood metabolites measured (*P* < 0.005, Table 2). Blood Alkaline phosphatase (ALP), glucose and urea were significantly higher in the CS and the RPP groups (*P* < 0.0001). In contrast, Aspartate amino-transferase (AST), Alanine amino-transferase (ALT) and Creatine kinase (CK) were significantly lower in the CS and the RPP groups (*P* < 0.05).

**Table 2:** Least square means of serum metabolites of Creole kids according to the dietary groups (Hay^1^, CS^2^, RPP^3^) infected or non-infected with an oral single dose of 10,000 third-larvae stage (L3) of *Haemonchus contortus*: Hay^1^; CS^2^; RPP^3^.

	Diets				*P*-value			
	Hay^1^	CS^2^	RPP^3^	SEM^4^	D^5^	T^6^	I^7^	D × T × I^8^
Alkaline Phosphatase, U/L	125.90^a^	317.91^b^	396.36^b^	37.32	0.0001	0.97	0.76	0.93
Gamma-Glutamyl-Transférase, U/L	36.95	42.11	36.98	3.34	0.5823	0.88	0.76	0.92
Aspartate Amino-Transferase, U/L	103.84^a^	76.75^b^	73.28^b^	5.43	0.0009	0.93	0.82	0.38
Alanine Amino-Transferase, U/L	34.11^a^	30.00^b^	29.36^b^	7.35	0.0122	0.01	0.86	0.19
Creatine Kinase, U/L	294.33^a^	168.28^b^	180.42^b^	23.21	0.0016	0.49	0.88	0.43
Creatinine, mg/L	9.15	11.67	9.75	0.99	0.2113	0.39	0.60	0.55
Glucose, g/L	0.46^a^	0.52^b^	0.56^b^	0.08	0.0001	0.001	0.79	0.09
Urea, g/L	0.34^a^	0.55^b^	0.52^b^	0.09	0.0001	0.009	0.36	0.58

Different superscripts indicate differences between least square means at *P* < 0.05.

^1^Hay distributed ad libitum.

^2^CS, Control Supplement, Hay distributed ad libitum + CS 2% of BW.

^3^RPP, Supplement enriched in Rumen Protected Proteins, Hay distributed ad libitum + RPP 2% of BW.

^4^SEM, Stan^d^ard Error of the Mean.

^5^D, *P*-valu^e^ of diets.

^6^T, *P*-value of the days post-infection.

^7^I, *P*-value of the infection status (infected vs non-infected).

^8^D × T × I, *P*-value of the interaction of the 3 fixed effects.

The amino acids composition of the duodenum-ileum contents of the animals was determined at 49 days post-infection (d.p.i.) after slaughtering (Table 3). No significant effect of the infection was observed (data not shown). The amounts (g/100 g DM) of the different amino acids were not different between the diets except for Ala and Pro which were higher in the supplemented groups (*P* < 0.05). The amounts of the total amino acids contents were also higher in the supplemented groups (*P* < 0.05). The composition (%) of the total amino acids were not different between the diets except for Asp, Thr, Glu, Tyr, Trp. Compared with RPP, the percentage of Thr and Tyr were higher in Hay and CS (*P* < 0.05). For Glu the percentage was higher in the RPP group (*P* < 0.05). The percentages of Asp were significantly higher in the CS group compared with the Hay and RPP groups (*P* < 0.05). The reverse was observed for Trp with higher percentages in the Hay and RPP groups compared with the CS groups (*P* < 0.05).

**Table 3:** Amino acids composition of the duodenum-ileum contents of Creole kids according to the diets: Hay; CS, RPP.

Amino Acids^1^ g/100 g DM (% of total AA)	Diets			
	Hay^2^	CS^3^	RPP^4^	*P values*
ASP	2.22 (9.6^a^)	2.83 (10.1^b^)	2.70 (9.6^a^)	0.13 (0.015)
THR	1.64 (7.1^a^)	1.92 (6.8^a^)	1.87 (6.5^b^)	0.49 (0.05)
SER	1.10 (4.7)	1.35 (4.8)	1.35 (4.8)	0.15 (0.67)
GLU	3.02 (13.1^a^)	3.76 (13.3^a^)	3.87 (13.8^b^)	0.19 (0.05)
GLY	1.22 (5.3)	1.44 (5.1)	1.47 (5.1)	0.41 (0.78)
ALA	1.24^a^ (5.4)	1.54^b^ (5.5)	1.58^b^ (5.7)	0.05 (0.31)
VAL	1.25 (5.4)	1.51 (5.3)	1.47 (5.2)	0.38 (0.34)
ILEU	1.03 (4.4)	1.26 (4.4)	1.24 (4.4)	0.38 (0.88)
LEU	1.85 (8.0)	2.26 (7.9)	2.33 (8.2)	0.27 (0.33)
TYR	1.28 (5.5^a^)	1.50 (5.3^a^)	1.37 (4.8^b^)	0.48 (0.05)
PHE	1.05 (4.5)	1.32 (4.7)	1.28 (4.6)	0.19 (0.15)
HIS	0.53 (2.3)	0.59 (2.2)	0.69 (2.5)	0.17 (0.51)
LYS	1.62 (7.0)	2.14 (7.5)	2.01 (6.9)	0.26 (0.2)
ARG	1.09 (4.7)	1.38 (4.8)	1.37 (4.6)	0.41 (0.85)
PRO	1.51^a^ (6.6)	1.81^b^ (6.6)	1.92^b^ (7.1)	0.05 (0.61)
MET	0.28 (1.2)	0.34 (1.2)	0.34 (1.2)	0.48 (0.99)
CYS	0.67 (2.9)	0.81 (2.9)	0.84 (3.1)	0.22 (0.68)
TRP	0.51^a^ (2.2^a^)	0.36^b^ (1.35^b^)	0.48^a^ (1.8^a^)	0.01 (0.01)
Total	23.1	28.1	28.2	0.05

Different superscripts indicate differences between least square means at *P* < 0.05.

^1^Amino acids, g/100 g of Dry Matter (DM) and (% of the total amino acids measured).

^2^Hay, Hay distributed *ad libitum*.

^3^CS, Control Supplement, Hay distributed ad libitum + CS 2% of BW.

^4^RPP, Supplement enriched in Rumen Protected Proteins, Hay distributed ad libitum + RPP 2% of BW.

The FEC and TFE were significantly higher in the Hay and the CS groups (Table 4, *P* < 0.05). The total number of adult nematodes (nematode burden) was significantly lower in the supplemented groups (CS and RPP, *P* > 0.05). No difference was observed between groups for the number of adult female nematodes (*P* > 0.05).

**Table 4:** Least squares means of the parasitological parameters of Creole kids according to the dietary groups (Hay^1^, CS^2^, RPP^3^) infected with an oral single dose of 10,000 third-larvae stage (L3) of *Haemonchus contortus*.

	Diets				
	Hay^1^	CS^2^	RPP^3^	SEM	*P* value
FEC^4^	9.08^a^	8.42^a^	7.56^b^	1.37	0.018^a^
TFE/day^5^	13.46^a^	13.06^a^	12.02^b^	1.17	0.013^a^
Male^6^	661^a^	211^b^	117^b^	345	0.013^b^
Female^7^	614	329	167	368	0.082
Nematode burden^8^	1276^a^	543^b^	296^b^	703	0.036^b^
Prolificacy^9^	3842^a^	8296^b^	5243^a^	768	0.048

Different superscripts indicate differences between least square means at *P* < 0.05.

^1^Hay distributed *ad libitum* non supplemented.

^2^CS, Control Supplement, Hay distributed *ad libitum* + CS 2% of BW.

^3^RPP, Supplement enriched in Rumen Protected Proteins, Hay distributed *ad libitum* + RPP 2% of BW.

^4^FEC, back-transformed faecal eggs counts (eggs/g of faeces).

^5^TFE/day, back-transformed total excreted faecal eggs per day.

^6^Male, total adult male nematodes.

^7^Female, total adult female nematodes.

^8^Nematode burden, total adult nematodes.

^9^Prolificacy, mean number of eggs produced per female adult nematode per day.

### Haematological and serological parameters

**Figure 2:**
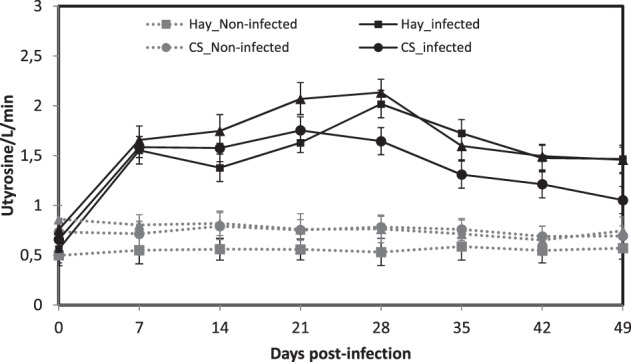
Least square means of serum pepsinogen in Creole kids according to the diets (Hay^1^, CS^2^, RPP^3^) infected with an oral single dose of 10,000 third-larvae stage (L3) of *Haemonchus contortus* or non-infected. ^1^Hay distributed *ad libitum* non supplemented. ^2^CS, Control Supplement, Hay distributed *ad libitum* + CS 2% of BW. ^3^RPP, Supplement enriched in Rumen Protected Proteins, Hay distributed *ad libitum* + RPP 2% of BW.

**Figure 3:**
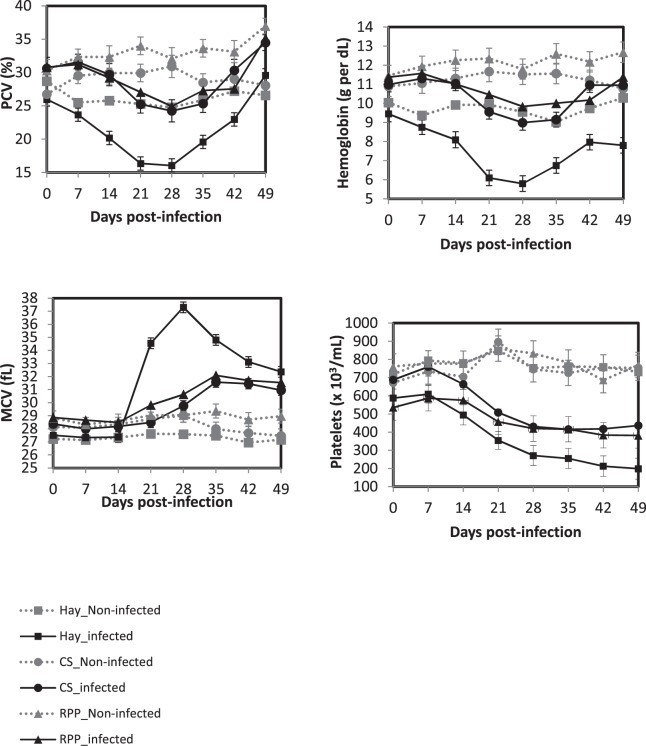
Least square means of haematological parameters in Creole kids according to the diets (Hay^1^, CS^2^, RPP^3^) infected with an oral single dose of 10,000 third-larvae stage (L3) of *Haemonchus contortus* or non-infected. ^1^Hay distributed *ad libitum* non supplemented. ^2^CS, Control Supplement, Hay distributed *ad libitum* + CS 2% of BW. ^3^RPP, Supplement enriched in Rumen Protected Proteins, Hay distributed *ad libitum* + RPP 2% of BW.

The levels of serum pepsinogen increased significantly in all infected groups at 7 d.p.i. to reach a plateau until 28 d.p.i. (*P* < 0.05, Fig. 2). Thereafter, the levels of serum pepsinogen decreased in all groups but remained significantly higher in the non-infected groups (*P* < 0.05). No effect of the diet was observed in the infected and the non-infected groups (*P* > 0.05). In contrast, for the PCV, the hemoglobin and the mean corpuscular volume (MCV), significant interaction was observed between the diet and the infection status (infected *vs*. non-infected, *P* < 0.01, Fig. 3). A transitory anemia observed only in the Hay groups from 14 until 28 d.p.i. (significant decrease of PCV and hemoglobin, *P* < 0.05) was correlated with an increase of the MCV (Fig. 3). In contrast, a thrombocytopenia was observed in all the infected animals but was significantly more important in the Hay group (*P* < 0.05).

**Figure 4:**
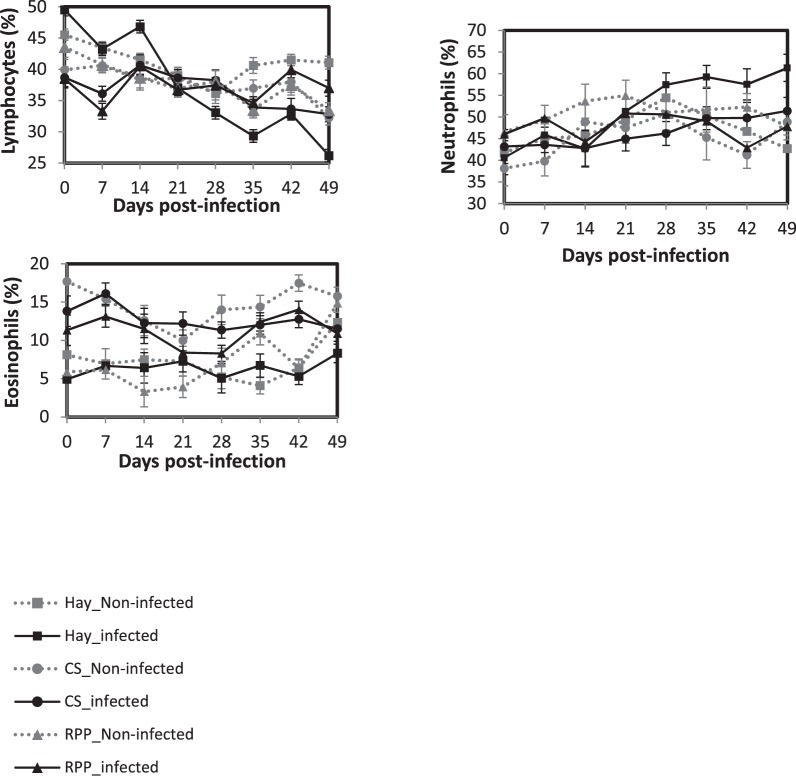
Least square means of blood immune cells in Creole kids according to the diets (Hay^1^, CS^2^, RPP^3^) infected with an oral single dose of 10,000 third-larvae stage (L3) of *Haemonchus contortus* or non-infected. ^1^Hay distributed *ad libitum* non supplemented. ^2^CS, Control Supplement, Hay distributed *ad libitum* + CS 2% of BW. ^3^RPP, Supplement enriched in Rumen Protected Proteins, Hay distributed *ad libitum* + RPP 2% of BW.

The percentage of circulating lymphocytes decreased slightly but significantly in infected animals (*P* < 0.01, Fig. 4). At 35 and 49 d.p.i. the percentage of circulating lymphocytes was significantly lower in the infected animals of the Hay group compared with the other groups infected and non-infected. A significant effect of the d.p.i. was observed for the percentage of neutrophils but no effect of the diet, the infection or their interaction was observed. The percentage of circulating eosinophils was higher in the CS group whatever the infection status (*P* < 0.05). No effect of the d.p.i., the infection status or their interaction was observed (*P* > 0.05). No significant effect was observed for the percentage of circulating basophile and monocytes (data not shown, *P* > 0.05).

**Figure 5:**
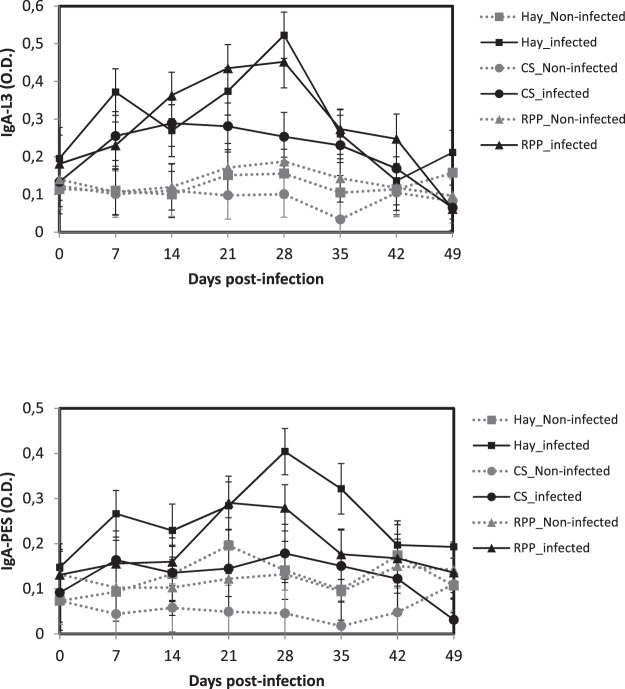
Least square means of serological IgA response against crude extract of L3 (anti-L3) antigens and against adults *Haemonchus contortus* excretion secretion products (anti-ESP) in Creole kids according to the diets (Hay^1^, CS^2^, RPP^3^) infected with an oral single dose of 10,000 third-larvae stage (L3) of *H. contortus* or non-infected. ^1^Hay distributed *ad libitum* non supplemented. ^2^CS, Control Supplement, Hay distributed *ad libitum* + CS 2% of BW. ^3^RPP, Supplement enriched in Rumen Protected Proteins, Hay distributed *ad libitum* + RPP 2% of BW.

Following the experimental infection with *H. contortus* the levels of IgA anti-L3 and anti-ESP responses increased in all the infected animals from 7 d.p.i. to peak between 21 and 28 d.p.i., and then decreased rapidly to reach a baseline at 42 d.p.i. to the end of the infection (*P* < 0.05, Fig. 5). Infected animals in the Hay and the RPP groups had IgA responses more pronounced than those of the CS group (*P* < 0.05). The level of IgA anti-PES was higher in animals of the Hay group at 28 and 35 d.p.i. (*P* < 0.05).

## Discussion

The positive effect of nutrient supplementation on the reduction of morbidity and mortality due to GIN infection in small ruminant has been described for a long time^12,13^. It is now hypothesized that host resistance to GIN is expected to arise from protein-rich rather than from energy-rich feeds^11^. However, since most of the studies used over-feeding of proteins to show host resistance to GIN, the adoption of this strategy as a non-chemical control method is not yet feasible from an economic or an environmental point of view. A fine-tuning of protein supplementation would be on the quality in term of amino acids contents. Indeed, it has been suggested that in case of metabolizable protein scarcity the effect of the quality of the feed on the resistance to GIN was significant^11^. Unfortunately, due to the ruminal activity, the manipulation of the amino acids profiles absorbed by the intestine is not an easy task. Indeed, in ruminants the proteins arriving in the intestine are a mix of dietary proteins that escaped from the microbial degradation in the rumen (i.e. by-pass proteins), microbial proteins synthetized from dietary amino acids and endogenous intestinal proteins^14^. The profile of the microbial proteins is stable, but that of by-pass proteins and the proportion of both depends on the composition of the feed^15,16^. Thus, the objective of this study was to investigate the effect of a supplement enriched in rumen-protected proteins on the resistance of Creole kid goats to *H. contortus*. Except for Glu, Tyr and Thr the profile of intestinal amino acids of animals fed with the RPP diet was not different from the CS and/or the Hay diet. Since the flow of amino acids arising from the abomasum is not homogenous over time, it is tempting to hypothesize that the delay between the slaughtering and the last supplement intake which was 24 h in this study, was probably too important to measure the impact of the diet on the amino acids profiles in the duodenum-ileum. In the same manner, supplementation has a significant effect on blood metabolites, with no specific effect for the RPP diet. In accordance with previous studies in ruminants, urea and glucose were higher in supplemented animals^17,18^. Alkaline phosphatase levels, which is an index of skeletal and antler growth in artiodactyls and associated with osteoblastic activity, were also higher in supplemented animals which showed the higher growth rate^19,20^. Aspartate Amino-Transferase, Alanine Amino-Transferase and Creatine Kinase levels were higher in the Hay groups showing the lower growth rate for the non-infected and weight loss for the infected ones. Such results would be markers of intense liver function to meet the energy and protein requirements for maintenance and production. These enzymes have been reported to be responsible for the protein balance during the lactation peak in dairy cattle and sheep^21–23^. No significant difference was observed between the Hay and the CS diets on the FEC and the TFEC. In accordance with a previous study in this biological model, the CS diet induced resilience (i.e. same level of parasitism with maintenance of production and physiological parameters) rather than resistance (i.e. decreased level of parasitism together with maintenance of production and physiological parameters) to *H. contortus* infection^24^. In the same manner, we showed that the CS diet reduced the severity and the lengthening of the regenerative anaemia and the thrombocytopenia induced by *H. contortus*.

The study showing that depletion of CD4+ T lymphocytes significantly increased the parasitic load in a resistant sheep breed was the first to demonstrate a close association between the resistance to GIN infection and the host immune response^25^. Thereafter, significant negative phenotypic correlation has been found between blood lymphocyte counts and *H. contortus* fecundity^26^. Our results are in accordance with a previous study in lambs showing that *H. contortus* induced lymphopenia^27^. In contrast with a previous study in Creole goat^28^, blood eosinophil counts were not statistically affected by the nutritional status. The high mean blood eosinophil counts in non-infected animals suggested that the one month parasite-free period was probably not enough to allow a significant decrease of the circulating eosinophils. In contrast with sheep, in goat the peripheral blood eosinophils would not play a key role in the protective response to GIN^25,29–33^.

Interestingly, the FEC and the TFEC were significantly lower in the RPP diet suggesting that this diet induced resistance to the experimental *H. contortus* infection. No difference was found between the two supplemented diets (CS *vs*.RPP) for the total number of nematodes, but the RPP diet reduced the prolificacy of the adult female nematodes. Here, the underlying mechanisms of resistance would not control the worm population establishment but rather the prolificacy of the adult female nematodes. The IgA response has been described as the major effector mechanism that control nematode egg production in sheep^34,35^. The strong association with GIN resistance and the favourable effects on growth have suggested that parasite-specific IgA might serve as a useful marker of resistance to infection^36^. In goats, the IgA response was positively correlated to FEC, suggesting that this response would be a marker of the level of parasitism^33,37^. In this study, the highest IgA responses were observed in animals with the highest nematode burden (i.e. the Hay compared with the CS diets). However, while the FEC and the TFEC were lower in animals fed with the RPP diet the IgA response were similar to those of the Hay diet. Our data suggested that the RPP diet would improve the goat IgA response to control nematode egg production as in sheep.

## Materials and Methods

All animal care, handling techniques, procedures as well as license for experimental infection and blood sampling were approved by the current law on animal experimentation and ethics (HC-69-2014-1 from the Animal Care and Use Committee of French West Indies and Guyana), according to the certificate number A-971-18-02 of authorization to experiment on living animals issued by the French Ministry of Agriculture, before the initiation of the experiment.

### Animals and experimental design

The experiment was conducted at the experimental unit of the INRA Antilles-Guyane research center (PTEA, Tropical Platform of the Experiment on the animal, 16°12′06.6″N 61°39′51.6″W). The Creole goats male kids (n = 48, 13.34 ± 2.4 kg body weight (BW); 4 months old) had experienced GIN infection at pasture before being randomly placed indoors in the individual pens corresponding to their experimental groups balanced according to their BW, 4 weeks before the experimental infection. The animals were drenched with levamisole (Polystrongle^®^, Coophavet, Ancenis, France, 8 mg/kg BW), toltrazuril (Baycox ovis^®^, Bayer healthcare, Lille, France, 20 mg/kg BW) and albendazole (Valbazen^®^ 1.9%, Zoetis, Paris, France, 7.5 mg/kg BW) and then were housed under worm-free conditions. During this period, nematode faecal egg counts (FEC) remained at zero. Each animal was placed under one of 3 distinct diets (n = 16 animals/diet): Hay (Hay *ad libitum* non supplemented), CS (Hay ad libitum + 2% of the BW of Control Supplement/kids), RPP (Hay ad libitum + 2% of the BW of Supplement enriched in Rumen Protected Proteins/kids) and had free access to fresh water. The supplements (CS and RPP) were distributed to the animals in form of pellets. The composition and nutritional values of the diets is shown in Table 1. After this 4 weeks period of adaptation to the diets and the individuals pens, a total of 10 animals/diet were experimentally infected with a single oral dose of 10,000 *H. contortus* third-stage infective larvae (L3) and 6 animals/diet remain non-infected: infected (I) and non-infected (NI) groups. The experiment was conducted for a total time of 77 days: 28 days before and 49 days after the experimental infection.

The L3 were obtained 48 days before the experimental infection from coproculture of monospecifically infected donor Creole goats with isolates previously obtained from Creole goats reared on pasture in different farms in Guadeloupe^38^.

### Animal samples and measurements

From the day of infection until the end of the experiment each animal was weighed weekly to adjust individually the offered quantities at 120% of the maximum intake capacity according to the BW changes and to measure individual growth rates. Blood samples were collected weekly by jugular venipuncture on each animal by using disposable syringes and 20-Ga needles in tubes containing an anticoagulant for complete blood counts (BD Vacutainer® spray-coated K3EDTA, Becton, Dickinson and Company, New Jersey, USA) and in dry tubes for serum analysis (BD Vacutainer^®^, Becton, Dickinson and Company, New Jersey, USA). Blood samples were analysed by an automaton (Melet Schloesing, MS9-5s, Osny, France). The number of circulating eosinophils was determined with Malassez cell counter^39^. Blood samples from each animal were centrifuged for 5 min. at 5000 rpm. Serum samples were then frozen at −20 °C until analysis. Serum pepsinogen levels were determined according to the method of Dorny and Vercruysse^40^. The biochemical parameters of the blood were determined by serum analysis (Melet Schloesing, MScan2, Osny, France). For FEC measurements during the experimental infection, approximately 10 g of faeces were collected in plastic tubes directly from the rectum of each animal, and transported from the experimental facility to the laboratory in refrigerated vials. The samples were individually analysed using a modified McMaster method for rapid determination and FEC was expressed as the number of eggs/g faeces^41^. The faeces were collected twice a day in collection bags that had been fitted to each animal and weighted. The total faecal eggs excreted per day (TFEC) were calculated as follow: TFEC = FEC (eggs per gram of faeces) × weight of total faeces excreted per animal per day (g). At slaughter (49 d.p.i), the contents of the abomasum of infected animals were collected individually. The abomasum was isolated with contents to determine the worm burden. The parasites were collected, counted and sorted according to the method of Bambou *et al*.^42^. We collected and lyophilized the duodenum contents in order to perform aminograms. Dry matter was determined for each sample and amino acids were assayed by HPLC after hydrolysis with 6 N hydrochloric acid at 110 °C for 23 h. Methionine and cystine underwent performic oxidation before hydrolysis. Tryptophan was hydrolysed with barite at 110 °C for 16 h. In general, the methods used for amino acids analyses were comparable with those described by Lahaye *et al*.^43^ or Cozannet *et al*.^44^ where more details are given. The levels of IgA anti-L3 and IgA anti-ESP was measured by indirect ELISA according to Bambou *et al*. (2008). Crude extracts of *H. contortus* L3 and excretory/secretory products of adults (ESP) were prepared according to Bambou, *et al*.^38^. In order to compare results between assays, a positive control consisting of a pool of sera containing IgA antibody was included on each plate, and OD450 of unknown samples were altered in proportion with changes of this standard. To measure the animal’s ingestion and digestibility, all feed offered and refused were individually weighed and sampled. During these periods, the whole of faeces excretions were individually measured and sampled. Daily, samples were proportionally pooled and preserved. The measures were realized during periods of 5 consecutive days. These measures were repeated during 8 measurements periods.

### Statistical Analysis

The parameters measured were analysed by a linear mixed model using the Proc Mixed of the software SAS (version 9.4 TS Level 1M3). Because of the skewed distributions, FEC, TFEC and eosinophils variables were logarithm transformed in Log (FEC + 15), Log (TFEC + 15) and log (eosinophils + 1) respectively to normalize the data. The other haematological and nutritional data were square-root-transformed to normalize residual variances. The model included fixed effects of time post-infection, diets, infection status, and the significant interaction. The comparisons between means were conducted by the least squares means procedure. The significancy was fixed at *P* ≤ 0.05 of probability.

## Data Availability

All data supporting the results of this study are included within this article.
